# Development of An Automatic Approaching System for Electrochemical Nanofabrication Using Visual and Force-Displacement Sensing

**DOI:** 10.3390/s120708465

**Published:** 2012-06-25

**Authors:** Lei-Jie Lai, Shi-Yu Zhou, Guo-Ying Gu, Li-Min Zhu

**Affiliations:** State Key Laboratory of Mechanical System and Vibration, School of Mechanical Engineering, Shanghai Jiao Tong University, Shanghai 200240, China

**Keywords:** approaching, contact detection, displacement-force sensing module, flexure mechanism, visual sensing module

## Abstract

In this paper, a fast automatic precision approaching system is developed for electrochemical nanofabrication using visual and force-displacement sensing. Before the substrate is fabricated, the template should approach the substrate accurately to establish the initial gap between the template and substrate. During the approaching process, the template is first quickly moved towards the substrate by the stepping motor until a specified gap is detected by the visual feedback. Then, the successive approach using the switch of macro-micro motion with a force-displacement sensing module is triggered to make the template contact with the substrate to nanometre accuracy. The contact force is measured by the force-displacement sensing module which employs the high-resolution capacitive displacement sensor and flexure compliant mechanism. The high sensitivity of this capacitive displacement sensor ensures high accuracy of the template-substrate contact. The experimental results show that the template can reach the substrate accurately and smoothly, which verifies the effectiveness of the proposed approaching system with the visual and the force-displacement sensing modules.

## 1. Introduction

Recently, various electrochemical nanofabrication methods [1–3] are widely developed to fabricate nano-metric structure due to their important advantages of high efficiency, feasibility for large areas, high resolution, and direct processability of solutions [4]. In the various electrochemical nanofabrication processes, the most important problems which limit their extensive use are the fine determination and adjustment of the distance between the template and the substrate. Although the nanopositioning stages equipped with the piezoelectric actuators and the capacitive displacement sensors have been developed to realize the fine adjustment of the gap distance [5], the fine control of the template-substrate distance is still a pendent problem due to the difficulty of accurate template-substrate contact detection [4]. Hence, it is important to make the template-substrate contact to nanometer accuracy and determine the initial gap and the zero point accurately before the substrate is fabricated.

Several methods have been proposed to solve this problem. A lock-in technique that uses force modulation [6] and lock-in amplifier [7] is employed to establish the template-substrate contact to nanometer accuracy.

In a nanoindentation tester, the slight change in the contact stiffness which is obtained by the slope of the linear fit of the proceeding and succeeding 30 data points from the force sensor is used to detect the template-substrate contact [8]. The force obtained by the deflection of the cantilevered stamp is used to detect the contact [9]. To improve the approach speed in atomic force microscope, an automatic approaching method using a Gaussian laser beam is adopted for decision making in determining mode transitions from fast motion to slow motion [10].

This paper presents a novel fast automatic approaching system using a visual sensing module and a high sensitive force-displacement sensing module. The force-displacement sensing module adopts the capacitive displacement sensor to measure the slight deformation of the flexure mechanism when the contact force is applied. Compared with other force sensors, this kind of sensing module possesses ultra high sensitivity to detect the contact force, and ensures high accuracy of the template-substrate contact. The successive approach driven by the switch of the macro-micro motion ensures that the contact only occurs at the micro motion process without any overshooting and crashing. Another advantage of this system is that the visual detection module is employed in the electrochemical nanofabrication for the coarse approach to shorten the time for the approaching process. The resolution of the visual sensing module can reach up to 20 *μ*m, which is higher than the previous works [10,11].

The remainder of the paper is organized as follows. The specific designs of the approaching system including the macro-micro dual driven positioning stage, the force and displacement sensing module and the visual sensing module are described in Section 2. The novel approaching method, which consists of two steps, namely the fast approach and the successive approach, is proposed in Section 3. The experimental setup and results are conducted in Section 4. Finally, Section 5 concludes the paper.

## 2. Overview of the Approaching System

**Figure 1.**
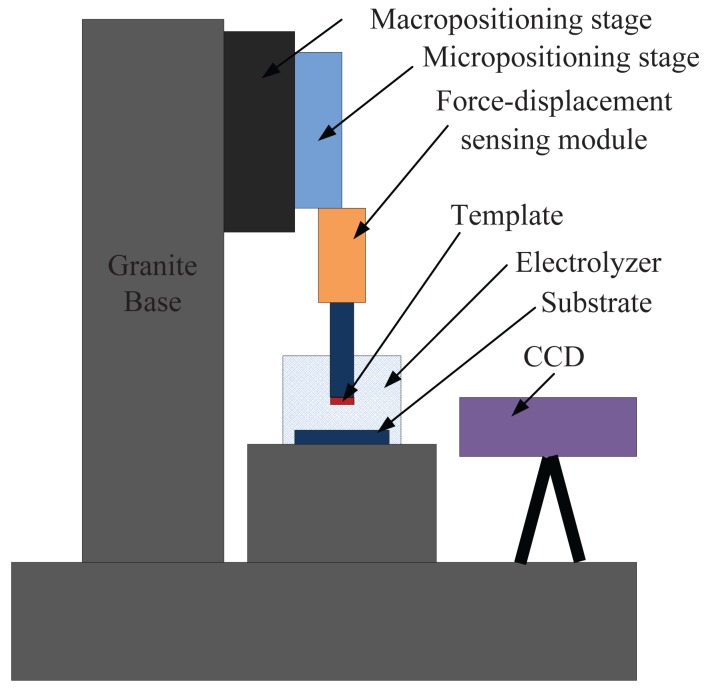
The schematic of the approaching system.

The automatic precision approaching system for electrochemical nanofabrication consists of a macro-micro dual driven positioning stage, a force-displacement sensing module and a visual sensing module. The overall system diagram is depicted in Figure 1. The macro-micro dual driven positioning stage, which includes the macropositioning stage and the micropositioning stage, controls the vertical movement of the template. The force-displacement sensing module fixed on the micropositioning stage is used to detect the contact force between the template and the substrate. The visual sensing module is employed to estimate the gap distance between the template and the substrate with a resolution in the order of tens of microns. The template is mounted on the moving platform of the sensing module and the substrate is fixed on the bottom of the electrolyzer. The whole system is mounted on a granite bridge base. The granite is a suitable material for stable structure due to its low thermal expansion coefficient, high stiffness and high damping ratio.

### 2.1. The Macro-Micro Dual Driven Positioning Stage

For the reduction of backlash and good holding stability, the macropositioning stage with 75 mm motion range uses a 100:1 harmonic-drive five-phase stepping motor, a pre-loaded ball screw, and a cross-roller guide. The pitch of the stage is 2 mm. The step angle of the harmonic geared stepping motor with 100:1 gear ratios is 0.0072°/step. The resolution of the macropositioning stage can be calculated as

(1) 0.0072(°/step)×(2/360)(mm/°)=40(mn/step).

The high resolution ensures that the macropositioning stage can move the micropositioning stage smoothly and stability. The harmonic geared reducer adopted in the macropositioning stage can be self-locking when the external force is applied to the macropositioning stage. Compared with the servo motor, the stepping motors have little random vibration at its rest position, and the positioning stability can well meet the nanometer precision requirements [12].

**Figure 2.**
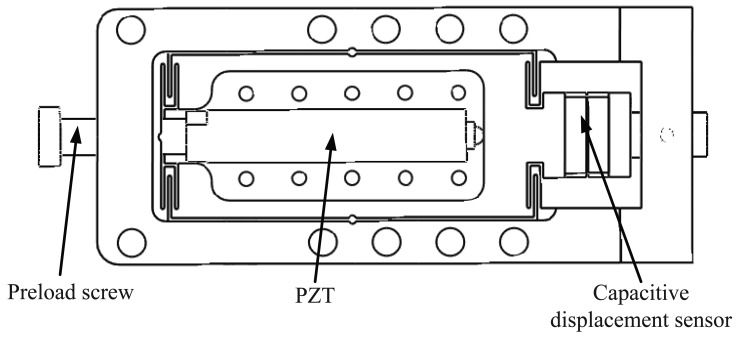
Structure of the micropositioning stage.

The micropositioning stage shown in Figure 2 is mounted on the macropositioning stage to obtain the macro-micro dual driven positioning stage. The motion range of the micropositioning stage is 40 *μ*m. The double compound parallelogram flexure is adopted for the micropositioning stage to remove the parasitic motions [13]. The PZT actuator is used to drive the flexure mechanism, and the displacement of the micropositioning stage is measured by the capacitive displacement sensor to realize the close-loop displacement control of the stage.

**Figure 3.**
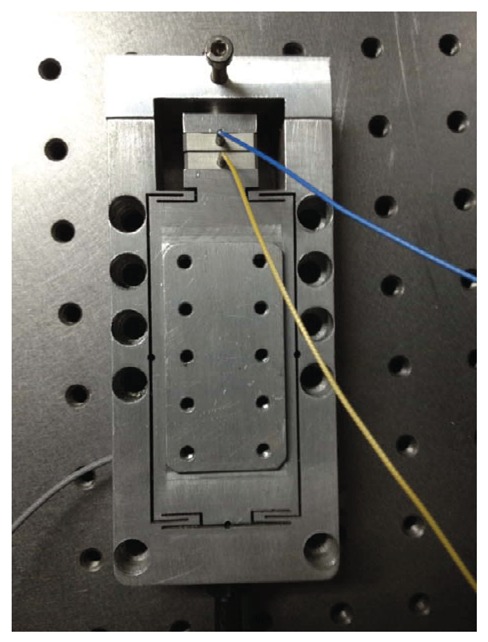
Photo of the micropositioning stage.

**Figure 4.**
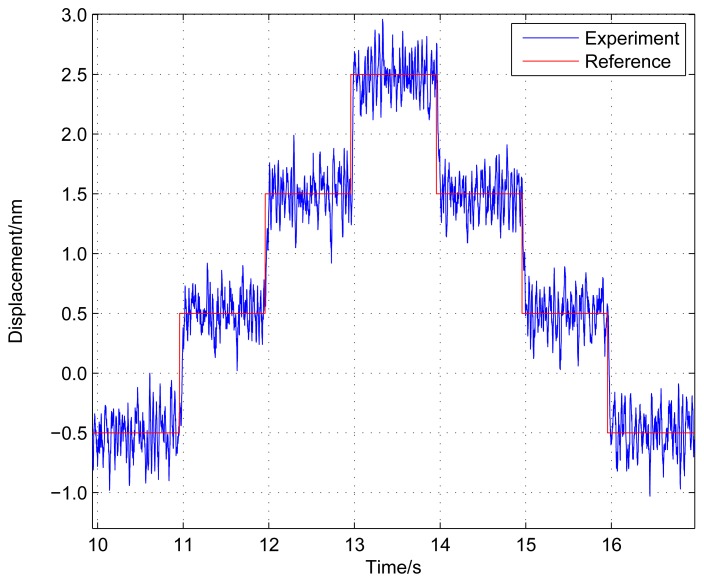
Positioning resolution of the micropositioning stage.

To obtain the positioning resolution of the stage, an experiment with a staircase input signal with 1 nm height is performed using the PID feedback controller on the machined stage shown in Figure 3. From the positioning experimental results shown in Figure 4, the positioning resolution of the stage can achieve sub-nanometer level.

### 2.2. The Force-Displacement Sensing Module

**Figure 5.**
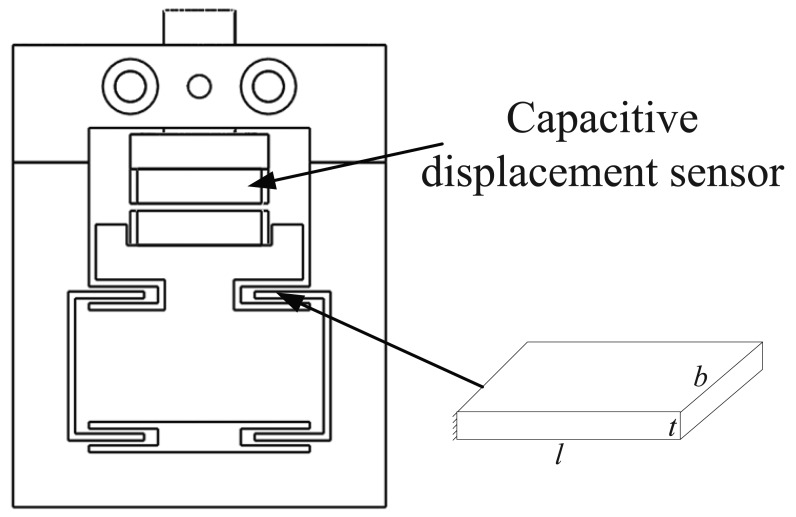
Structure of the force-displacement sensing module.

The structure of the force-displacement sensing module is shown in Figure 5. The double compound parallelogram flexure is also adopted for the sensing module. The capacitive displacement sensor detects the axial plastic deformation of the flexure platform in the approaching process to estimate the contact force between the template and the substrate. The axial stiffness and the first resonant frequency of the structure can be calculated as follows [14]:

(2) $$K=\frac{2Ebt^{2}}{l^{3}};f=\frac{1}{2\pi }\sqrt{\frac{K}{M}}$$

where *l* = 8 *mm, b* = 20 *mm* and *t* = 0.7 *mm* are the detailed dimensions of the flexure, as shown in Figure 5. The sensing module is made of Al7075, whose Young's modulus (E) is 71 Gpa. From the Equation (2), the designed stiffness is 0.1876 g/nm, and the first resonance frequency is 1,050 Hz, high enough to reject external disturbances.

**Figure 6.**
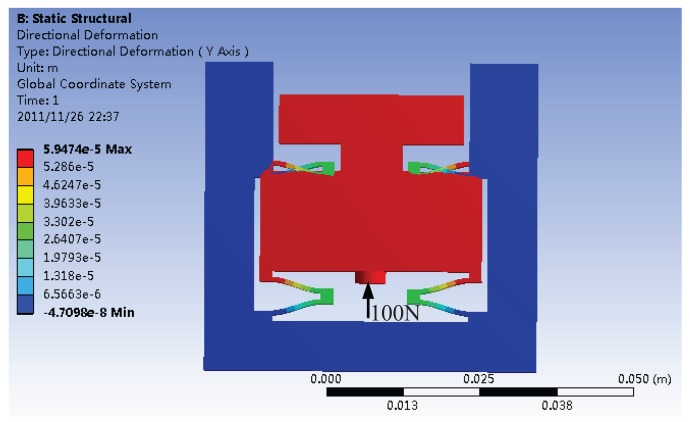
Static deformation under 100 N force.

The ANSYS Workbench finite element software is employed to estimate the compliance of the structure. When a force of 100 N is applied to the structure, the static deformation is shown in Figure 6. The compliance can be calculated as the ratio of the maximum displacement of the platform to the applied force.

**Figure 7.**
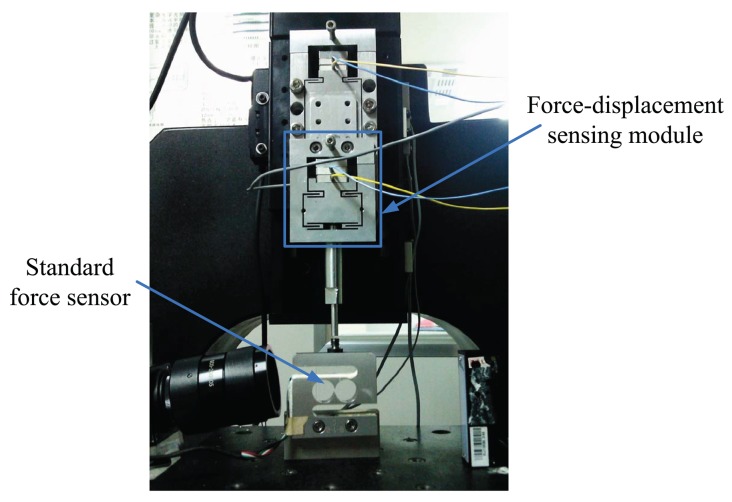
Calibration device of the sensing module.

**Figure 8.**
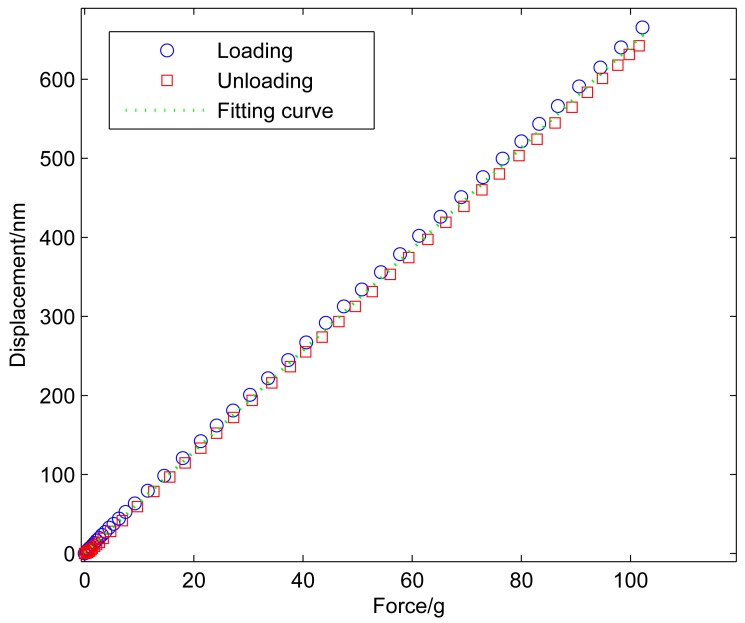
Calibration results of the sensing module.

Experimental calibration is also conducted by the standard force sensor to acquire the relationship between the force and the displacement of the sensing module, as shown in Figure 7. In the experimental calibration, the macropositioning stage moves the sensing module with the step of 40 nm to change the applied force gradually. The force of the standard force sensor and the displacement of the capacitive displacement sensor are simultaneously recorded after each step. As shown in Figure 8, the compliance can be obtained using the least square fitting method. The compliances of this module calculated from the analytical model, the FEA and the experimental results are compared in Table 1. The three results agree well. The resolution of the capacitive sensor is in the order of picometers in theory [15], and limited by the stray radiation, electronic induced noise and geometric effects. Hence, the force resolution of the sensing module is below micronewton in the ideal environment.

### 2.3. The Visual Sensing Module

**Figure 9.**
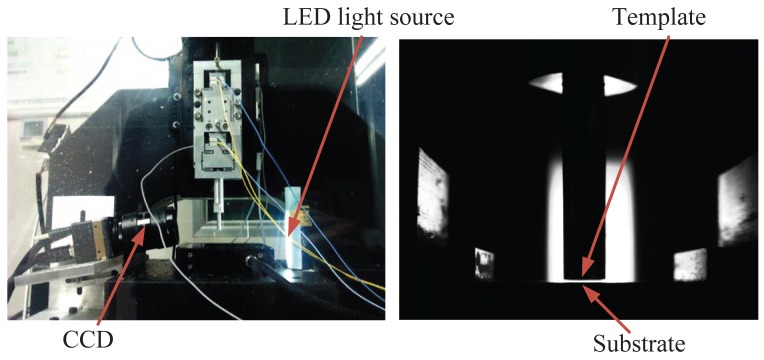
The visual sensing system.

In order to estimate the template-substrate distance, a visual sensing module is mounted beside the template and the substrate, as shown in Figure 9. A CCD camera with a pixel size of 7.4 *μ*m and a telecentric lens with a large optical zoom and depth of field realize the physical resolution of 46.25 *μ*m/pixel for the visual module. A LED light source is selected as a secondary light source to improve the image quality and ensures the accuracy and stability of the visual module. The gap distance between the template and the substrate is estimated by detecting the contour of the template and the substrate by an image processing software. The resolution of the visual module can be increased up to 23 *μ*m/pixel by using the Zernike moment-based subpixel edge detection method [16].

**Figure 10.**
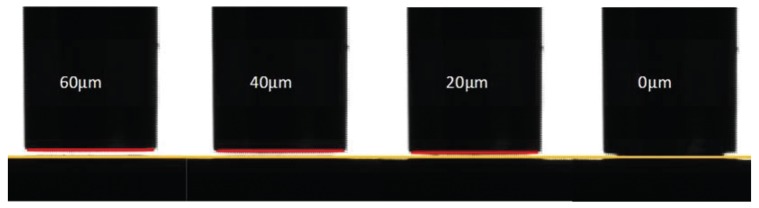
Images with different gap.

Experimental calibration is conducted to quantify the narrow gap distance detected by the visual sensing system. First, the template is manually approached to the substrate with the help of the visual and force-displacement sensing modules. By adjusting the displacement of the micropositioning stage precisely, the template can contact with the substrate with no contact force. Then, the macropositioning stage moves the template upwards by 20 *μ*m, and the number of pixels between two edges which is equivalent to the gap of 20 *μ*m is estimated by the image processing software. Using the same method, the pixels' numbers for the gaps of 40 *μ*m and 60 *μ*m are calculated as well. A series of images with different gap are shown in Figure 10.

## 3. The New Approaching Method

**Figure 11.**
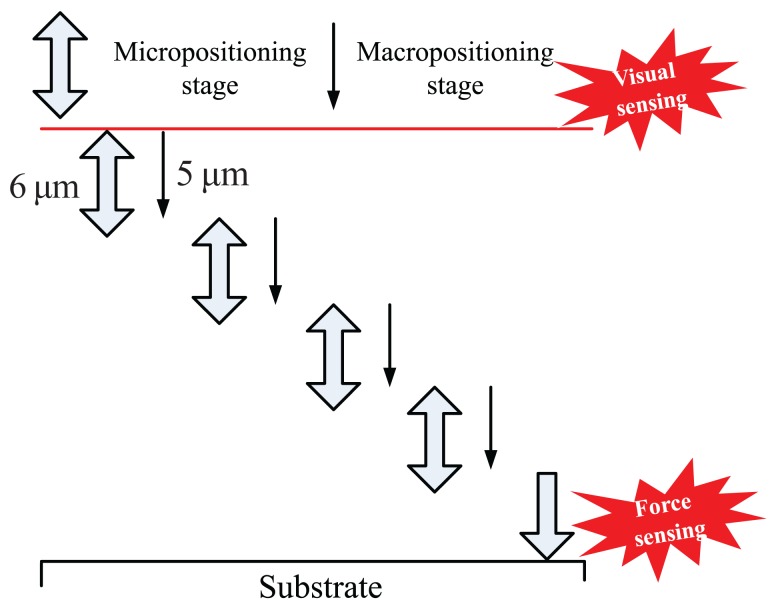
The approaching method using visual and force-displacement sensing.

The template-substrate approach is achieved in two steps: the fast approach driven by the macropositioning stage with the visual sensing feedback and the successive approach using the macro-micro dual driven stage, as shown in Figure 11.

In the fast approaching step, the macropositioning stage first moves the template towards the substrate at a high speed until the gap between the template and the substrate is within 200 *μ*m with the help of a visual sensing feedback from the visual sensing module. When the detected gap is less than 200 *μ*m, the approaching speed is reduced by 5 times. The fast approaching step ends when a gap of less than 50 *μ*m is detected by the visual module. The visual module provides an effective criterion for determining the safe distance to enhance the approaching speed in the fast approaching step. With this method, the template can be approached to the substrate from a great distance to tens of microns in a short time without damaging to the template and the substrate.

In the successive approaching step, the micropositioning stage first moves the template towards the substrate for 6 *μ*m at a speed of 1 nm/ms to see if the contact is detected. When there is no response from the force-displacement sensing module, the micropositioning stage is retracted, and the macropositioning stage extends five more microns, so on and so on. When the template encounters the substrate, the output of the force-displacement sensing module starts to increase from zero. Once the detected contact force is greater than the threshold value that is determined based on the material properties and processing characteristics, the successive approaching step finishes.

To determine the zero point for the nanofabrication, the micropositioning stage is then retracted until the detected contact force decreases back to zero so that the contact deformations of the force-displacement sensing module and the substrate are released. The current position point can be determined as the processing zero point. The successive approaching method ensures the template reaches the substrate without overshooting and crashing.

## 4. Experimental Setup and Results

**Figure 12.**
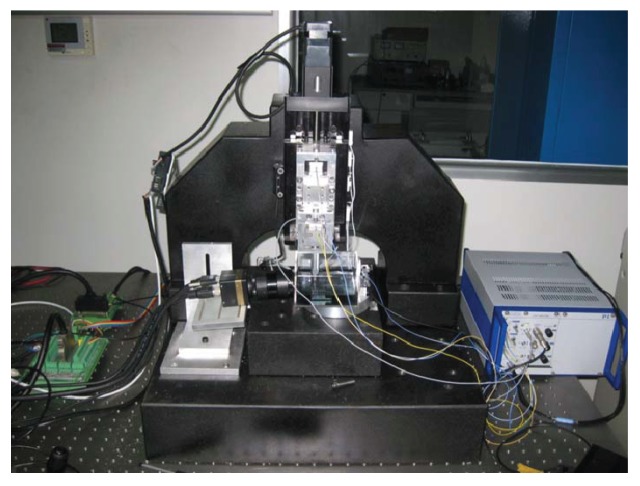
Experimental setup of the whole system.

Figure 12 shows the whole experiment setup for the approaching system. A motion control card (PCI-4P from HIWIN) is used to send pulses to the stepping motor. A PZT (P-840.30 from PI) is adopted to drive the micropositioning stage through a PZT amplifier (E-503.00 from PI). Two capacitive displacement sensors (D-100.00 from PI) are used to measure the displacements of the micropositioning stage and the force-displacement sensing module respectively. In order to improve the approaching resolution and positioning accuracy, a high-resolution data acquisition card (PCI-9524 from ADLINK) equipped with 24-bit A/D and 16-bit D/A converters is used to acquire the voltage of the capacitive sensors (0–10 V) and to apply the control voltage (0–10 V) to the PZT amplifier.

The approaching experiment was conducted at the threshold of 20 nm for the deformation of the sensing module (corresponding to the 3.12 g of contact force). It is worth mentioning that the threshold of the contact force can be reduced by increasing the compliance of the sensing module with the same deformation. The resolution of the force-displacement sensing module can be calculated as the ratio of the noise level from the capacitive displacement sensor to the structure compliance of the sensing module. The force resolution can be improved by increasing the compliance of the sensing module with the same noise level. Increasing the resolution and sensitivity of the force sensing module can result in the reduction of the force threshold and improve the contact accuracy effectively. Moreover, the large force threshold may cause damages to the template and substrate [17]. Therefore, it will be better to reduce the threshold to improve the accuracy of the contact detection and prevent damages to the template and substrate.

**Figure 13.**
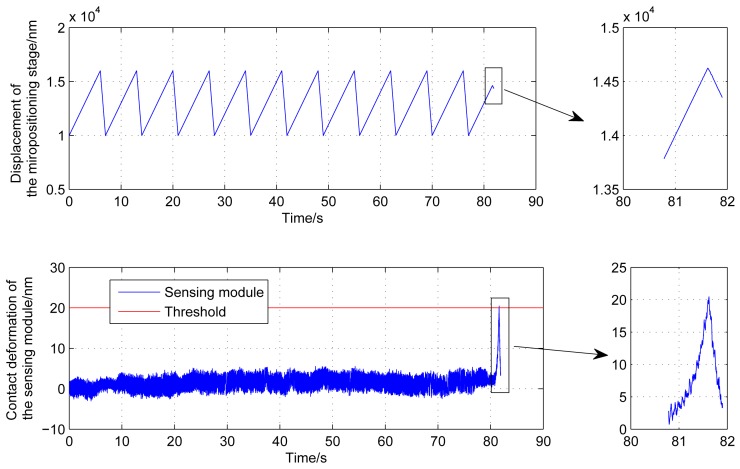
The experimental results of the approaching process.

As shown in Figure 13, after a successive approaching driven by the macro-micro positioning stage, the template starts to contact with the substrate, and the output of the force-displacement sensing module quickly increases up to the threshold value. The micropositioning stage is then retracted to determine the zero point. However, the capacitive displacement sensor can be easily influenced by the system electronic noise, ambient conditions, *etc.* [18]. The noise and drift are the two main uncertainties in force measurement, which can result in wrong determination of the zero point. The successive approaching process may fail to finish accurately. Figure 13 shows that the drift and the noise level of the sensing module are both less than 5 nm in the approaching process. Although the two values are very small, the approaching accuracy can be influenced. Therefore, 50 data points are acquired from the force-displacement sensing module to estimate the drift and noise values before the contact is detected. The mean value and standard deviation of the 50 data points are calculated respectively. The sum of the mean value and standard deviation (3 nm in this experiment) is taken as the new threshold for the determination of the zero point when the micropositioning stage is retracted, as shown in Figure 13. From the experimental results, the retracted displacement of micropositioning stage is larger than the threshold deformation of the sensing module to compensate for the template deformation and assembly errors in the contact process. As shown in Figure 13, all the transitions between the macropositioning stage and the micropositioning stage have no dynamic effect on the force sensing module, which effectively demonstrates the stability of the proposed method. In the whole successive approaching step, the micropostioning stage is always under the closed-loop operation, which has fast response time to put the template into feedback quickly and safely, even in the transitions between the two stages. It can be seen that the template-substrate contact can be achieved within nanometer range, and the template can reach the substrate smoothly and quickly.

## 5. Conclusions

In this paper, an automatic approaching system is presented for electrochemical nanofabrication with a visual sensing and a force-displacement sensing modules. The approaching process can be divided into two phases: the fast approach and the successive approach. The fast approach is driven by the macropositioning stage under the guidance of the the visual sensing module. The visual sensing system provides a effective decision making criterion for the switching from the fast approach to the successive approach. The high resolution of the visual sensing module effectively shortens the approaching time. The successive approach is conducted by the switch of macro-micro motion. The high-resolution capacitive displacement sensor is employed to measure the elastic axial deformation of the flexure mechanism in the force-displacement sensing module when the contact force is applied. The high sensitivity of capacitive displacement sensor ensures that the approaching accuracy can achieve the nanometer level. The experimental results show that excellent performances of the approaching system are achieved. The practical significance of this approaching system for the electrochemical nanofabrication is concluded as follows:

- The approaching process is automatically accomplished by the programmed control without any human operation, which can increase the efficiency and throughput of the electrochemical nanofabrication.
- The visual sensing module employed in the electrochemical nanofabrication instrument for the determination of the safe distance helps to prevent damages to the template and substrate at the fast approaching step.
- The ultra-high accuracy capacitive displacement sensors used in the micropositioning stage and the force-displacement sensing module can greatly increase the accuracy of contact detection, and improve the reproducibility and fidelity of the electrochemical nanofabrication.

## Figures and Tables

**Table 1.** Comparison of the compliance of the sensing module.

	**Analytical Results**	**FEA Results**	**Test Results**
Compliace of the module (nm/g)	5.33	5.95	6.41
